# A relationship among the blood serum levels of interleukin‐6, albumin, and 25‐hydroxyvitamin D and frailty in elderly patients with chronic coronary syndrome

**DOI:** 10.1002/agm2.12201

**Published:** 2022-02-27

**Authors:** Jing‐rong Dai, Jie Li,, Xu He,, Hong Huang,, Yan Li

**Affiliations:** ^1^ School of Medicine Kunming University of Science and Technology Kunming China; ^2^ Department of Geriatrics The First People's Hospital of Yunnan Province Kunming China

**Keywords:** 25‐hydroxyvitamin D, albumin, chronic coronary syndrome, comprehensive geriatric assessment, coronary artery disease, elderly people, frailty, interleukin‐6

## Abstract

**Background:**

With the aggravation of the aging of the world population, frailty has become one of the common complications in elderly people. Its diagnosis is not objective, the pathogenesis is not clear, and interventions are not sound, thus intensifying the problem. Furthermore, frailty is closely associated with the occurrence and poor prognosis of coronary atherosclerotic heart disease. Moreover, few studies report on the prevalence of frailty in elderly patients with the chronic coronary syndrome (CCS).

**Objective:**

We aimed to investigate the prevalence of frailty in elderly patients with CCS. We analyzed the correlation between the blood serum levels of interleukin‐6 (IL‐6), albumin (Alb), and 25‐hydroxyvitamin D (25(OH)D) with frailty in elderly patients with CCS. We have also provided recommendations for helping the objective diagnosis as well as proposed new intervention methods in the future.

**Methods:**

Two hundred eight‐eight inpatients (≥60 years) with the chronic coronary syndrome were recruited at the Department of Geriatrics, the First People's Hospital of Yunnan Province, China. General information and laboratory examination data were collected. The comprehensive geriatric assessment was conducted via an internet‐based platform of the Comprehensive Geriatric Assessment (inpatient version) developed by us, among which frailty was assessed by the Chinese version of Fried Frailty Phenotype, a component of the assessment scale.

**Results:**

Among the total number of old patients with CCS, 87 (30.2%) had no frailty, 93 (32.3%) had early frailty, and 108 (37.5%) had frailty. According to the multivariate logistic regression analysis, after adjusting for confounding factors, IL‐6 (OR = 1.066, 95% CI 1.012–1.127), Alb (OR = 0.740, 95% CI 0.560–0.978), and 25(OH)D (OR = 0.798, 95% CI 0.670–0.949) were independently associated with frailty in the three groups of models.

**Conclusion:**

IL‐6 proved to be a risk factor for frailty in elderly patients with CCS, while Alb and 25(OH)D were protective factors, which make the potential targets for predicting and intervening frailty in elderly patients with CCS.

## Graphical abstract

With the aggravation of the aging of the world population, frailty has become one of the common complications in elderly people. Its diagnosis is not objective, the pathogenesis is not clear, and interventions are not sound, thus intensifying the problem. Furthermore, frailty is closely associated with the occurrence and poor prognosis of coronary atherosclerotic heart disease. Moreover, few studies report on the prevalence of frailty in elderly patients with the chronic coronary syndrome (CCS). We aimed to investigate the prevalence of frailty in elderly patients with CCS. We analyzed the correlation between the blood serum levels of interleukin‐6 (IL‐6), albumin (Alb), and 25‐hydroxyvitamin D (25(OH)D) with frailty in elderly patients with CCS. We have also provided recommendations for helping the objective diagnosis as well as proposed new intervention methods in the future. Two hundred eighty‐eight inpatients (≥60 years) with the chronic coronary syndrome were recruited at the Department of Geriatrics, the First People's Hospital of Yunnan Province, China. General information and laboratory examination data were collected. The comprehensive geriatric assessment was conducted via an internet‐based platform of the Comprehensive Geriatric Assessment (inpatient version) developed by us, among which frailty was assessed by the Chinese version of Fried Frailty Phenotype, a component of the assessment scale. Among the total number of old patients with CCS, 87 (30.2%) had no frailty, 93 (32.3%) had early frailty, and 108 (37.5%) had frailty. According to the multivariate logistic regression analysis, after adjusting for confounding factors, IL‐6 (OR = 1.066, 95% CI 1.012–1.127), Alb (OR = 0.740, 95% CI 0.560–0.978), and 25(OH)D (OR = 0.798, 95% CI 0.670–0.949) were independently associated with frailty in the three groups of models. IL‐6 proved to be a risk factor for frailty in elderly patients with CCS, while Alb and 25(OH)D were protective factors, which make the potential targets for predicting and intervening frailty in elderly patients with CCS.

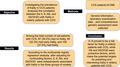

## 1 INTRODUCTION

With an in‐depth understanding of the pathophysiology and dynamic changes in the course of coronary artery disease, the ESC (European Society of Cardiology) published the ESC guidelines for the diagnosis and management of chronic coronary syndromes (CCSs), and the concept of CCSs was formally proposed. It refers to the different stages of coronary heart disease other than the clinical presentation dominated by acute coronary thrombosis.^1^ It further emphasized that the steady‐state period of non‐acute coronary syndromes was relative and could progress to acute coronary syndromes at any time.

Frailty is a special state in which the physical functions of elderly people gradually decline. It is characterized by weakened muscle strength and endurance, decreased physiological functions, increased vulnerability, decreased anti‐stress ability with subsequent adverse consequences such as disability, cognitive impairment, malnutrition, mental abnormalities, and even death.^2^, ^3^ Some studies have shown that frailty patients with coronary artery disease (CAD) were more likely to have adverse events than non‐frailty patients.^4^ In addition, in elderly CAD patients, longer life expectancy leads to more focus on improving patients' functional ability and quality of life.^5^ Therefore, frailty has attracted increasing attention as a way to identify adverse outcomes in patients with coronary artery disease.^6^ With the aging of the world population, the problem of frailty in old age is becoming increasingly prominent. To screen old adults at high risk of frailty, Fried et al. proposed the use of clinical phenotypes to characterize frailty, which consisted of five body components, including decreased muscle strength, reduced walking speed, fatigue, reduced physical activity, and unconscious weight loss.^7^ Nowadays, these criteria are widely used in clinics for the diagnosis of frailty.

Unfortunately, they do not provide an objective diagnostic basis, clear pathogenesis, and sound intervention methods. Studies have shown a high prevalence of senile frailty, and there was a close relationship among CGA and various serum indicators and frailty.^8^, ^9^, ^10^ Similarly, our previous study found that disabled 25‐hydroxyvitamin D (25(OH)D) and interleukin‐6 (IL‐6) were independent influencing factors of frailty in patients with stable chronic diseases.^11^ However, the studies on the prevalence and related factors of frailty in elderly patients with CCS were scarce. Moreover, there is a lack of objective biological markers for the diagnosis of frailty. Here, we aim to investigate the prevalence of frailty in elderly patients with CCS. Our main focus is on the correlation between the blood levels of IL‐6, albumin (Alb), and 25(OH)D with frailty in elderly patients with CCS. Establishing such a relation would be a good and solid theoretical basis for the objective diagnosis of frailty in elderly CCS patients as well as for proposing some interventions for improving the condition.

## 2 MATERIALS AND METHODS

### 2.1 Study design and participants

This is a cross‐sectional study conducted in Yunnan province, China. A total number of 288 elderly inpatients at the age of 60 years and above, diagnosed with CCS, were recruited at the Department of Geriatrics, the First People's Hospital of the Yunnan Province, China, from November 2019 to October 2021. The inclusion criteria were: (1) hospitalized review patients, aged ≥60 years, living in the Yunnan province for ≥3 years, without new diseases; (2) patients with no communication barriers and able to cooperate in the comprehensive geriatric assessment (CGA); (3) patients who were voluntarily participating in the study and have signed the informed consent; (4) people with diagnosed CCS according to the criteria of the 2019 ESC guidelines for the diagnosis and management of the chronic coronary syndrome. Following these guidelines the patients enrolled in the study were: (4.1) elderly people with suspected CAD and "stable" angina symptoms, regardless of dyspnea; (4.2) patients with newly emerging heart failure or left ventricular dysfunction suspected of CAD; (4.3) patients with asymptomatic or stable symptoms within 1 year after ACS or patients who recently underwent revascularization; (4.4) patients whose initial diagnosis or revascularization were done more than one year ago; (4.5) patients with angina pectoris, suspected vasospasm, or microcirculation diseases; (4.6) asymptomatic patients with coronary heart disease found during screening. The applied exclusion criteria were: (1) patients with Vitamin D supplementation, with albumin blood products, and antiinflammatory drugs in the past one month; (2) patients who were diagnosed with acute infectious diseases recently; (3) patients with serious physical and/or mental diseases and with communication barriers, who were unable to complete the CGA; (4) patients who were bedridden or unstable for a long time.

### 2.2 Data collection and measurement

#### 2.2.1 General demographic data collection

Patients’ information (derived from the inpatient electronic medical record system), including age, gender, body mass index (BMI, kg/m^2^), education level, vision, hearing, marital status, sleep time, current smoking (referring to smoking in the last 30 days before the survey), current drinking (referring to the alcohol consumption in the last 30 days before the survey), number of chronic diseases (including chronic obstructive emphysema, asthma, chronic cor pulmonale, chronic respiratory failure, chronic heart failure, coronary heart disease, hypertension, heart valve disease, chronic gastritis, peptic ulcer, chronic enteritis, chronic hepatitis, liver cirrhosis; chronic nephritis, chronic renal failure; hyperthyroidism, hypothyroidism; diabetes, gout, osteoporosis, rheumatoid arthritis, systemic lupus erythematosus, ankylosing spondylitis, osteoarthritis), and polypharmacy (oral drugs ≥5) were collected.

#### 2.2.2 Data collection of comprehensive geriatric assessment

Elderly patients were evaluated by trained geriatricians using the CGA system software. It was independently developed by the Department of Geriatrics, First People's Hospital of Yunnan Province, China and was applied in the current study. It represents an intelligent evaluation system composed of multiple universal assessment scales. Evaluators collected patients’ data through a WeChat mini‐program or computer. After the evaluation, the system automatically calculated scores and gave diagnosis reports. The assessment included patients’ nutritional status, evaluated by the short‐form mini‐nutritional assessment (MNA‐SF).^12^ Values ≥24.0 were considered as indicators of good nutrition, between 17.0 and 23.5 were designated as potential malnutrition, and <17.0 were classified as malnutrition. Patients’ cognitive function assessment was also done following the mini‐mental state examination(MMSE),^13^ where the system automatically classified the criteria according to the education level of the patients and obtained the conclusion, where values between 0 and 9 were classified as a severe impairment, between 10 and 20 were classified as moderate impairment, between 21 and 26 were classified as mild impairment, while scores between 27 and 30 were designated as normal cognitive functions. Patients' evaluation of anxiety and depression followed the 15‐item geriatric depression scale (GDS‐15),^14^ where scores ≥6 indicated anxiety and depression. The patients’ daily living ability assessment was according to the basic activities of daily living (BADL) scale,^15^ where scores between 91 and 100 were indicators of good daily living function, between 61 and 90 were regarded as mild functional impairment, between 41 and 60 were labelled as moderate functional impairment, between 21 and 40 were considered as severe functional impairment, whereas patients with scores between 0 and 20 were grouped as completely disabled. The sleep status assessment was done according to the Athens insomnia scale (AIS),^16^ where scores between 0 and 3 indicated good sleep, between 4 and 6 spoke for potential insomnia, whereas between 7 and 24 indicated insomnia. The fall risk assessment was according to the Morse fall scale (MFS),^17^ where scores between 0 and 24 classified the patients at low risk of fall, between 25 and 44 categorized the patients at moderate risk, whereas scores equal and above 45 categorized the elderly people at severe risk. The balance and gait function evaluation was following the performance‐oriented mobility assessment (POMA),^18^ where scores <15 indicated the risk of falling, between 15 and 24 designated balance dysfunction, whereas scores greater than or equal to 24 indicated good physical function. The visual analogue scale (VAS) was used for pain evaluation.^19^ Scores equal to 0 indicated lack of pain, between 1 and 3 designated mild pain, between 4 and 6 showed the presence of moderate pain, whereas between 7 and 10 indicated presence of severe pain. The evaluation of urinary incontinence was in harmony with the incontinence questionnaire simple form (ICI‐Q‐SF),^20^ where scores equal to 0 were classifying the patients into the group of asymptomatic urinary incontinence, between 1 and 7 were determining the elderly people with mild urinary incontinence, between 8 and 14 indicated moderate urinary incontinence, whereas the scores between 15 and 21 indicated that the patients had severe urinary incontinence. The constipation was assessed using the Roma III scale (≥2),^21^ while the Fried Scale^7^ was used for frailty assessment, as shown in Table 1.

**TABLE 1:** Contents of the Chinese version of Fried scale for frailty assessment

Serial number	Item	Details
1	Consciously fatigue	Fatigue >3 days in the past 1 week
2	Muscle strength decline	Grip strength: male < 23 kg, female < 14 kg
3	Decreased body function	Step speed (6‐meter walking experiment) time ≥7 s or speed <0.65 m/s
4	Weight loss	Unconscious weight loss of more than 3 kg or 5% of total body weight within 1 year
5	Activity reduction	Weekly activity <600 MET‐min/W (obtained through IPAQ)

Note

Lack of frailty assessment items was considered as no frailty, patients with 1~2 were diagnosed as pre‐frailty, whereas 3 were frailty.

IPAQ, International Physical Activity Scale.

#### 2.2.3 Collection of patients' blood serum biological indicators

Forty milliliters of fasting venous blood was collected from the hospitalized elderly patients from 6:00 to 8:00 am and sent to the clinical laboratory of the First People's Hospital of Yunnan Province, China for testing. The automatic analyzer Xiang Instrument L1550 was used for blood samples analyses. The blood was centrifuged at 1360 *g* for 5 min. The detected parameters included white (WBC) and red blood cells count (RBC), hemoglobin (Hb), platelets (PLT), and neutrophils count (NEUT), as well as the C‐reactive protein (CRP). The aspartate (AST) and alanine aminotransferase (ALT) were detected by the rate method. Triacylglycerols (TG) were detected by the deionization glycerol method, the total protein (TP) was detected by the biuret method, albumin (ALB) was detected by the bromocresol green method, while the total cholesterol (TC) was detected by the cholesterol oxidase method. High density (HDL) and low‐density lipoproteins (LDL) were detected by the elimination method. Creatinine (Cr) and glycosylated hemoglobin (HbA1c) were assayed by enzyme reactions. Urea nitrogen (BUN) was assayed by the urease UV rate method. Uric acid (UA) was assayed by enzyme calorimetry. Thyroid‐stimulating hormone (TSH), thyroid hormone (T4), ferritin, Vitamin B_12,_ folic acid, 25(OH)D, estradiol, testosterone, homocysteine (Hcy), fasting insulin (FINS) were detected by electrochemiluminescence. Tumor necrosis factor (TNF), IL‐10, IL‐6, IL‐12P70, IL‐1 and IL‐8 were detected by chemiluminescence.

### 2.3 Data quality control

Data quality control was done on two steps: (1) The Geriatrics Department of the First People's Hospital of Yunnan Province organized on‐site and internet training courses for the physicians taking part in elderly patients assessment on "Promotion and Application of Intelligent CGA System," focusing on learning the meaning of the questionnaire content and the way of inquiry. (2) The form of the “WeChat Mini Program” electronic questionnaire was uniformly adopted, and the investigator needed to ask the patient or an escort who was familiar with the basic situation of the patient throughout the whole process. (3) During the field investigation, errors were corrected and made up for omissions in time. Meanwhile, paper report forms were printed out for the completed scale promptly by computer and reviewed the report results. Before the patient's discharge, errors or missing data were corrected and completed.

### 2.4 Statistical methods

SPSS 23.0 software was used for statistical analysis. The measurement data (χ ± S) conforming to the normal distribution were expressed, and the comparison between the three groups was performed by one‐way analysis of variance. Measurement data of non‐normal distribution were expressed as M (P25, P75), and the Kruskal‐Wallis test was used for comparison between multiple groups. Counting data were expressed in relative numbers and the χ2 test was used for comparison between groups. Multivariate logistic regression analysis was used to analyze the influencing factors of frailty in elderly patients with CCS. *p* values <0.05 were considered as a statistically significant difference. To assess the relative contribution of these related factors to vulnerability, we conducted an analysis based on three different models.

### 2.5 Ethical consideration

Following the Declaration of Helsinki, this study was implemented after approval of the Medical Ethics Committee of the First People's Hospital of Yunnan Province (No. KHLL2021–KY034). All participants provided written informed consent to participate in the study.

## 3 RESULTS

### 3.1 General comparison among the demographic data of elderly patients’ in different frailty stages diagnosed with CCS

According to the Fried scale, the study population was divided into non‐frailty, pre‐frailty, and frailty groups, as shown in Table 2. Among the 288 patients, 87 (30.2%) had no frailty, 93 (32.3%) had pre‐frailty, and 108 (37.5%) had frailty. In our study, 111 patients (38.5%) were no more than 75 years old, between 75 and 85 years old were 92 (31.9%), and aged equal and above 85 years old were 82 (29.5%). What's more, the prevalence of visual and hearing impairment was 201 (69.8%) and 173 (60.1), respectively. The average number of chronic diseases suffered by the subjects was 7.72 ± 3.39, and there were 185 (64.2%) patients with polypharmacy (≥5 species). In addition, patients' age generally increased with the increase of the severity of frailty, namely the older the patients were, the higher the estimated risk of frailty (*p* < 0.001). Our results show that patients with impaired hearing and vision were more likely to have frailty (*p* < 0.05). Elderly patients with CCS were prone to a variety of chronic diseases, and a higher risk of frailty (*p *= 0.001) was estimated for the patients with more chronic diseases. In addition, patients with polypharmacy (taking ≥5 oral drugs) had a higher incidence of frailty than those without polypharmacy (*p *= 0.001).

**TABLE 2:** Comparison of the general demographic data of elderly patients diagnosed with CCS in different frailty states

Variable	Overall (*n* = 288)	Non‐Frailty (*n* = 87)	Pre‐Frailty (*n* = 93)	Frailty (*n* = 108)	χ2(F) value	*P* value
Age (years)^a^						
<75 years old	111 (38.5)	50 (67.8)	37 (39.8)	15 (13.9)	67.501	<0.001
≥75, <85 years old	92 (31.9)	24 (27.6)	35 (37.6)	33 (30.6)		
≥85 years old	82 (29.5)	4 (4.6)	21 (22.6)	60 (55.6)		
Gender^b^						
Male	173 (60.1)	48 (55.2)	56 (60.2)	69 (63.9)	1.527	0.466
Female	115 (39.9)	39 (44.8)	37 (39.8)	39 (36.1)		
BMI, mean ± *SD* ^a^	23.28 ± 4.14	23.63 ± 3.41	23.42 ± 5.54	22.87 ± 3.15	0.897	0.409
Education level^b^						
Illiteracy	12 (4.2)	1 (1.1)	6 (6.5)	5 (4.6)	7.599	0.269
Primary school	155 (53.8)	51 (58.6)	44 (47.3)	60 (55.6)		
Middle school	66 (29.9)	15 (17.2)	26 (28.0)	25 (23.1)		
College degree and above	55 (19.1)	20 (23.0)	17 (18.3)	18 (16.7)		
Vision condition^b^						
Normal	87 (30.2)	24 (27.6)	39 (41.9)	24 (22.2)	9.617	0.008
Decline	201 (69.8)	63 (72.4)	54 (58.1)	84 (77.8)		
Hearing condition^b^						
Normal	115 (39.9)	48 (55.2)	41 (44.1)	26 (24.1)	20.417	<0.001
Decline	173 (60.1)	39 (44.8)	52 (55.9)	82 (75.9)		
Marital status^b^						
Married	222 (77.1)	72 (82.8)	74 (79.6)	76 (70.4)	4.667	0.097
Divorced/Widowed	66 (22.9)	15 (17.2)	19 (20.4)	32 (29.6)		
Eating habits^b^						
Light diet mainly	248 (86.1)	71 (81.6)	82 (88.2)	95 (88.0)	2.114	0.347
Mainly salty and greasy diet	40 (13.9)	16 (18.4)	11 (11.8)	13 (12.0)		
Sleeping time (h)^a^, mean ± *SD*		6.74 ± 1.69	7.08 ± 1.78	7.19 ± 2.09	1.459	0.234
Current smoking status^b^						
No	224 (77.8)	65 (74.7)	76 (81.7)	83 (76.9)	1.363	0.506
Yes	64 (22.2)	22 (25.3)	17 (18.3)	25 (23.1)		
Current drinking situation^b^						
No	242 (84.0)	68 (78.2)	82 (88.2)	92 (85.2)	3.529	0.171
Yes	46 (16.0)	19 (21.8)	11 (11.8)	16 (14.8)		
Number of chronic diseases (species)^a^, mean ± *SD*	7.72 ± 3.39	6.70 ± 3.59	7.46 ± 3.45	8.75 ± 4.23	7.297	0.001

ANOVA.

Chi‐square test.

Fisher's Exact test.

### 3.2 Comparison of geriatric syndrome data of elderly patients with CCS in different frailty states

Among the 288 patients, 89 (30.9%) had potential malnutrition and 62 (21.5%) had severe malnutrition; 98 (34.0%) had mild cognitive impairment, 49 (17.0%) had moderate dysfunction, and 14 (4.9%) had severe cognitive impairment; 141 (49.0%) had anxiety and depression; 75 (26.0%) had mild daily dysfunction, 68 (23.6%) had moderate daily dysfunction, and 5 (1.7%) had severe daily dysfunction; 55 (19.1%) had potential insomnia and 108 (37.5%) had insomnia; 165 (57.3%) had low risk of falling, 56 (19.4%) had moderate risk of falling, and 67 (23.3%) had high risk of falling; 87 (30.2%) had balance dysfunction and 68 (23.6%) at risk of falling; 71 (24.7%) had chronic pain. At the same time, patients with good nutritional status demonstrated a lower risk of frailty, while patients with potential malnutrition or severe malnutrition had a higher proportion (*p* < 0.05). A higher risk of frailty (*p* < 0.005) was observed in elderly patients with more severe impairment of their cognitive function, daily living ability, sleep status, and balanced gait. Patients with anxiety, depression, and chronic pain were more prone to frailty (<0.05). See Table 3 for details.

**TABLE 3:** Comparison of geriatric syndrome data in elderly patients with CCS in different frailty states

Variable	Overall (*n* = 288)	Non‐Frailty (*n* = 87)	Pre‐Frailty (*n* = 93)	Frailty (*n* = 108)	χ^2^(F) value	*P* value
Nutritional status^b^						
Good nutrition	137 (47.6)	53 (60.9)	47 (50.5)	37 (34.3)	15.224	0.004
Potential malnutrition	89 (30.9)	22 (25.3)	28 (30.1)	39 (36.1)		
Severe malnutrition	62 (21.5)	12 (13.8)	18 (19.4)	32 (29.6)		
Cognitive function^b^						
Normal cognitive function	127 (44.1)	62 (71.3)	39 (41.9)	22 (20.4)	56.432	<0.001
Mild cognitive impairment	98 (34.0)	20 (23.0)	35 (37.6)	47 (43.5)		
Moderate cognitive impairment	49 (17.0)	5 (5.7)	15 (16.1)	29 (26.9)		
Severe cognitive impairment	14 (4.9)	0 (0.0)	4 (4.3)	10 (9.3)		
Anxiety and depression^b^						
No	147 (51.0)	59 (67.8)	47 (50.5)	41 (38.0)	17.198	<0.001
Yes	141 (49.0)	28 (32.2)	46 (49.5)	67 (62.0)		
Daily living ability^b^						
Good life function	140 (48.6)	53 (60.9)	49 (52.7)	38 (35.2)	39.541	<0.001
Mild dysfunction	75 (26.0)	23 (26.4)	30 (32.3)	22 (20.4)		
Moderate dysfunction	68 (23.6)	9 (10.3)	12 (12.9)	47 (43.5)		
Severe dysfunction	5 (1.7)	2 (2.3)	3 (2.2)	1 (0.9)		
Sleep condition^b^						
Sleep well	125 (43.4)	51 (58.6)	36 (38.7)	38 (35.2)	13.065	0.011
Potential insomnia	55 (19.1)	15 (17.2)	18 (19.4)	22 (20.4)		
Insomnia	108 (37.5)	21 (24.1)	39 (41.9)	48 (44.4)		
Fall risk situation^b^						
Low risk	165 (57.3)	73 (83.9)	52 (55.9)	40 (37.0)	51.219	<0.001
Moderate risk	56 (19.4)	12 (13.8)	20 (21.5)	24 (22.2)		
High risk	67 (23.3)	2 (2.3)	21 (22.6)	44 (40.7)		
Balance and gait function^b^						
No abnormality	133 (46.2)	65 (74.7)	45 (48.4)	23 (21.3)	62.374	<0.001
Balance disorder	87 (30.2)	17 (19.5)	30 (32.3)	40 (37.0)		
Fall risk	68 (23.6)	5 (5.7)	18 (19.4)	45 (41.7)		
Chronic pain^b^						
No	217 (75.3)	73 (83.9)	70 (75.3)	74 (68.5)	6.144	0.046
Yes	71 (24.7)	14 (16.1)	23 (24.7)	34 (31.5)		
Urinary incontinence^b^						
No	255 (88.5)	81 (93.1)	85 (91.4)	95 (88.0)	1.595	0.450
Yes	33 (11.5)	6 (6.9)	8 (8.6)	12 (12.0)		
Constipation^b^						
No	216 (75.0)	73 (83.9)	65 (69.9)	78 (72.2)	5.420	0.067
Yes	72 (25.0)	14 (16.1)	28 (30.1)	30 (27.8)		
Polypharmacy (kind) (≥5 species)^b^						
No	103 (35.8)	44 (50.6)	33 (35.5)	26 (24.1)	14.734	0.001
Yes	185 (64.2)	43 (49.4)	60 (64.5)	82 (75.9)		

ANOVA.

Chi‐square test.

Fisher’s Exact test.

### 3.3 Comparison of blood serum biomarkers in elderly patients with CCS in different frailty states

A plethora of blood serum biomarkers have been studied in elderly patients with different frailty stages. They were thoroughly described in the Materials and Methods section together with the methods used for analysis. Our results showed that the levels of WBC, Hb, Alb, TC, and 25‐(OH)D decreased with the severity of frailty. Interestingly, the WBC count and Hb concentration in the frailty group blood serum were lower than those in the pre‐frailty group, while the latter was lower than those in the non‐frailty group (*p* < 0.05). The levels of CRP, Cr, BUN, E2, Vitamin B_12_ (Vit B_12_), TNF, Hcy, and IL‐6 increased with the aggravation of frailty (*p* < 0.05). See Table 4 for details.

**TABLE 4:** Comparison of serum biomarkers in elderly patients with CCS in different frailty states

Variable	Overall (*n* = 288)	Non‐Frailty (*n* = 87)	Pre‐Frailty (*n* = 93)	Frailty (*n* = 108)	*Z*(*F*)value	*P* value
WBC ^a^(×10^9^/L), mean ± *SD*	6.73 ± 2.70	6.49 ± 2.06	6.85 ± 3.20	6.82 ± 2.70	0.499	0.608
RBC ^a^(×10^12^/L), mean ± *SD*	4.34 ± 0.74	4.59 ± 0.60	4.47 ± 0.63	4.02 ± 0.81	18.479	<0.001
Hb ^a^(g/L), mean ± *SD*	132.34 ± 23.42	140.89 ± 21.10	134.24 ± 19.45	123.82 ± 25.53	14.497	<0.001
PLT ^a^(×10^9^/L), mean ± *SD*	206.88 ± 78.09	207.49 ± 68.24	218.50 ± 90.22	196.38 ± 73.38	2.024	0.134
NEUT ^c^(×10^9^/L), [M(P_25_,P_75_)]	4.02 (2.87, 5.07)	3.81 (2.73, 4.89)	3.91 (2.84, 5.21)	4.14 (3.00, 5.24)	1.588	0.452
CRP ^c^(mg/L), [M(P_25_,P_75_)]	4.65 (1.08, 23.87)	1.50 (0.51, 9.26)	3.19 (0.96, 19.20)	13.65 (2.99, 38.19)	31.940	<0.001
AST ^c^(U/L), [M(P_25_,P_75_)]	19.00 (15.00, 25.75)	20.00 (16.00, 24.00)	19.00 (15.00, 22.37)	19.00 (15.00, 27.00)	1.143	0.565
ALT ^c^(U/L), [M(P_25_,P_75_)]	14.50 (10.00, 21.75)	16.00 (11.00, 24.00)	15.00 (10.00, 20.00)	13.00 (9.00, 23.00)	4.629	0.099
TG^a^ (mmol/L)	1.44 ± 0.96	1.59 ± 0.88	1.45 ± 1.07	1.30 ± 0.90	2.269	0.105
TC, ^a^(mmol/L), mean ± *SD*	4.12 ± 1.09	4.28 ± 1.15	4.20 ± 1.00	3.90 ± 1.09	3.324	0.037
TP ^a^(g/L), mean ± *SD*	64.37 ± 7.49	65.88 ± 6.30	64.02 ± 7.15	63.47 ± 8.49	2.678	0.070
Alb ^a^(g/L), mean ± *SD*	36.03 ± 5.70	38.60 ± 4.48	36.30 ± 6.06	33.73 ± 5.34	20.150	<0.001
HDL ^a^(mmol/L), mean ± *SD*	1.06 ± 0.33	1.10 ± 0.32	1.10 ± 0.33	1.00 ± 0.33	2.918	0.056
LDL ^a^(mmol/L), mean ± *SD*	2.45 ± 0.87	2.55 ± 0.94	2.49 ± 0.77	2.33 ± 0.88	1.743	0.177
Cr ^c^(μmol/L), [M(P_25_,P_75_)]	78.00 (63.00, 96.00)	70.00 (60.00, 85.00)	77.00 (60.50, 95.50)	86.50 (66.00, 103.00)	11.053	0.004
BUN ^c^(μmol/L), [M(P_25_,P_75_)]	6.45 (5.10, 8.68)	5.80 (4.90, 7.00)	6.50 (5.25, 9.25)	7.30 (5.50, 9.57)	14.776	0.001
UA ^a^(μmol/L)	375.26 ± 123.52	360.75 ± 93.66	386.08 ± 108.72	377.63 ± 153.08	0.977	0.378
HbA1c ^c^(%), [M(P_25_,P_75_)]	6.25 (5.68, 6.77)	6.25 (5.77, 7.31)	6.31 (5.68, 6.92)	6.09 (5.60, 6.53)	3.801	0.149
TSH ^c^(mU/L), [M(P_25_,P_75_)]	2.62 (1.58, 4.46)	2.60 (1.63, 4.46)	3.02 (1.65, 4.46)	2.42 (1.41, 4.46)	0.845	0.656
T_3_ ^c^(nmol/L), [M(P_25_,P_75_)]	1.10 (0.82, 1.48)	1.10 (0.90, 1.55)	1.10 (0.81, 1.59)	1.05 (0.66, 1.35)	5.313	0.070
T_4_ ^a^(nmol/L), mean ± *SD*	79.44 ± 18.71	78.93 ± 14.38	80.88 ± 18.30	78.61 ± 21.97	0.411	0.663
Ferritin ^c^(ng/ml), [M(P_25_,P_75_)]	276.50 (128.52, 395.76)	295.49 (185.30, 330.39)	228.27 (100.94, 364.54)	329.21 (127.37, 513.62)	4.851	0.088
Vit B_12_ ^c^(pmol/L), [M(P_25_,P_75_)]	402.50 (253.25, 552.00)	330.00 (231.00, 490.00)	363.00 (236.00, 519.50)	486.00 (324.25.00, 746.50)	17.467	0.014
Folate^c^(nmol/L), [M(P_25_,P_75_)]	15.65 (10.03, 21.68)	18.00 (12.80, 21.50)	15.00 (10.05, 22.35)	13.80 (8.55, 21.63)	4.003	0.135
25‐(OH)D ^a^(ng/ml), mean ± *SD*	20.74 ± 8.20	24.04 ± 8.23	20.55 ± 8.37	18.24 ± 7.10	13.119	<0.001
Estradiol ^a^(pmol/L)	118.36 ± 56.34	104.12 ± 50.34	118.84 ± 58.44	129.39 ± 57.04	4.982	0.007
Testosterone ^c^(nmol/L), [M(P_25_,P_75_)]	6.57 (0.53, 12.01)	7.45 (0.49, 11.79)	6.48 (0.57, 14.39)	5.24 (0.56, 11.54)	0.207	0.902
Hcy ^c^(μmol/L), [M(P_25_,P_75_)]	18.22 (14.13, 23.58)	16.90 (12.40, 19.20)	18.10 (14.80, 21.45)	19.35 (16.23, 33.33)	21.040	<0.001
FINS ^c^(μU/ml), [M(P_25_,P_75_)]	7.18 (4.31, 9.32)	8.16 (4.96, 11.74)	6.25 (4.23, 8.75)	7.24 (4.03, 8.46)	5.571	0.062
TNF ^c^(pg/ml), [M(P_25_,P_75_)]	9.00 (4.84, 21.18)	6.55 (4.18, 10.47)	10.47 (5.19, 23.72)	10.47 (5.35, 35.09)	17.418	<0.001
IL‐10 ^c^(pg/ml), [M(P_25_,P_75_)]	4.66 (3.57, 6.16)	4.49 (3.51, 6.15)	4.75 (3.70, 6.16)	4.74 (3.33, 6.16)	1.023	0.600
IL‐6 ^c^(pg/ml), [M(P_25_,P_75_)]	17.69 (7.75, 28.91)	8.35 (5.95, 25.17)	19.62 (10.98, 33.67)	23.64 (12.25, 33.58)	28.711	<0.001
IL‐12P70 ^c^(pg/ml), [M(P_25_,P_75_)]	5.45 (3.88, 6.46)	5.68 (4.35, 6.46)	5.33 (3.53, 6.46)	5.39 (3.95, 6.46)	1.495	0.474
IL‐1ß ^c^(pg/ml), [M(P_25_,P_75_)]	4.98 (3.74, 8.01)	5.09 (3.83, 8.01)	5.09 (3.84, 7.97)	4.85 (3.54, 8.01)	1.037	0.595
IL‐8 ^c^(pg/ml), [M(P_25_,P_75_)]	24.46 (13.08, 59.39)	18.91 (11.34, 59.39)	28.92 (13.22, 59.39)	25.89 (13.76, 59.39)	2.658	0.265

25(OH)D, 25 hydroxyvitamin D; ALB, albumin; ALT, alanine aminotransferase; AST, aspartate aminotransferase; BUN, Urea Nitrogen; Cr, creatinine; CRP, C reactive protein; FINS, fasting insulin; Hb, hemoglobin; HbA1c, glycosylated hemoglobin; Hcy, homocysteine; HDL, high‐density lipoprotein; IL, Interleukin; LDL, low‐density lipoprotein; NEUT, neutrophil fraction; PLT, platelet count; RBC, red blood cell count; T3, triiodothyronine; T4, thyroid hormone; TC, total cholesterol; TG, triacylglycerol; TP, total protein; TSH, Thyroid Stimulating Hormone; UA, uric acid; WBC, white blood cell count.

*p < 0.05.

**p < 0.001.

ANOVA.

Chi‐square test.

Fisher’s Exact test.

### 3.4 Regression analysis of influencing factors in elderly patients with CCS frailty

To further look at possible interrelation among IL‐6, Alb, 25(OH)D, and frailty in elderly people with CCS, we conducted a multivariate logistic regression analysis and the results are shown in Table 5. Three models have been drawn based on the conducted multivariate logistic regression analysis. Model 1 used a multiple logistic regression adjusted for IL‐6, Alb,25(OH)D, RBC, Hb, CRP, TC, Cr, BUN, Vit B_12_, hcy, E2, and TNF. Model 2 relied on a multivariate logistic regression in sync with Model 1 and the geriatric syndrome (chronic pain, nutritional status, cognitive function, anxiety and depression, daily living ability, insomnia, risk of falling, balance, gait, and polypharmacy). Model 3 included a multiple logistic regression adjustment conducted according to Model 1, Model 2, and certain demographic data (age, vision, hearing, and number of chronic diseases). After adjusting for the above confounding factors, IL‐6 (OR = 1.037, 95% CI 1.008–1.068), Alb (OR = 0.868, 95% CI 0.776–0.971), and 25(OH)D (OR = 0.937, 95% CI 0.884–0.992) were independently associated with pre‐frailty. After adjusting for the above confounding factors, IL‐6 (OR = 1.049, 95% CI 1.007–1.093), Alb (OR = 0.789, 95% CI 0.789–0.975), and 25(OH)D (OR = 0.813, 95% CI 0.694–0.953) were independently associated with frailty in the three groups of models.

**TABLE 5:** Relationship among IL‐6, Alb, 25(OH)D, and frailty in elderly patients with CCS

Variable	Mode 1				Mode 2				Mode 3			
	Pre‐frailty		Frailty		Pre‐frailty		Frailty		Pre‐frailty		Frailty	
	Versus Non‐Frailty											
	OR (95% CI)	*P* value	OR (95% CI)	*P* value	OR (95% CI)	*P* value	OR (95% CI)	*P* value	OR (95% CI)	*P* value	OR (95% CI)	*P* value
IL‐6	1.037 (1.011, 1.064)	0.005	1.045 (1.016, 1.075)	0.002	1.033 (1.005, 1.062)	0.022	1.037 (1.003, 1.073)	0.034	1.037 (1.008, 1.068)	0.014	1.049 (1.007, 1.093)	0.023
Alb	0.893 (0.82, 0.974)	0.011	0.867 (0.787, 0.956)	0.004	0.883 (0.793, 0.983)	0.023	0.833 (0.714, 0.972)	0.020	0.868 (0.776, 0.971)	0.013	0.789 (0.638, 0.975)	0.028
25(OH)D	0.939 (0.897, 0.984)	0.008	0.916 (0.859, 0.976)	0.007	0.933 (0.882, 0.986)	0.014	0.893 (0.801, 0.995)	0.041	0.937 (0.884, 0.992)	0.025	0.813 (0.694, 0.953)	0.011
Hcy	1.03 (0.992, 1.070)	0.127	1.051 (1.006, 1.097)	0.025	1.046 (0.995, 1.101)	0.079	1.049 (1.003, 1.097)	0.038	1.051 (0.996, 1.110)	0.071	1.071 (0.997, 1.152)	0.062
TNF	1.027 (1.004, 1.050)	0.021	1.031 (1.006, 1.056)	0.015	1.021 (0.997, 1.045)	0.084	1.061 (1.011, 1.113)	0.016	1.011 (0.988, 1.034)	0.36	1.038 (0.999, 1.078)	0.054
Vtm B12	1.001 (1, 1.002)	0.036	1.003 (1.002, 1.005)	<0.001	1.002 (1.000, 1.003)	0.011	1.003 (1.000, 1.005)	0.049	1.002 (1.000, 1.003)	0.018	1.003 (0.999, 1.007)	0.178
Balance and Gait Function						0.311		0.014		0.633		0.032
Balance disorder					1.598 (0.604, 4.230)	0.345	0.039 (0.004, 0.366)	0.004	1.486 (0.537, 4.110)	0.446	0.008 (0.000, 0.366)	0.013
Risk of falling					2.919 (0.631, 13.504)	0.171	0.108 (0.009, 1.251)	0.075	1.982 (0.304, 12.942)	0.475	0.054 (0.001, 2.080)	0.117
Risk of falling						0.023				0.062		0.699
Medium risk					2.247 (0.688, 7.338)	0.180	1.812 (0.274, 11.973)	0.537	2.028 (0.602, 6.826)	0.254	1.128 (0.098, 13.027)	0.923
High risk					10.202 (1.74, 59.807)	0.01	11.951 (1.448, 98.603)	0.021	7.974 (1.274, 49.909)	0.026	4.035 (0.159, 102.329)	0.398
Age										0.028		0.010
≥75, <85 years old									1.631 (0.590, 4.514)	0.346	2.824 (0.369, 21.590)	0.317
≥85 years old									13.486 (2.006, 90.667)	0.007	973.59 (11.312, 83795.266)	0.002
Number of chronic diseases									1.123 (0.972, 1.296)	0.115	1.232 (0.890, 1.706)	0.209

CI, confidence interval; OR, odds ratio.

### 3.5 Correlational analyses of frail elderly patients with CCS

Spearman rank correlation analysis showed that Alb (r_s_ = −0.366, *p *< 0.001) and 25(OH)D (r_s_ = −0.275, *p *< 0.001) were negatively correlated with frailty in elderly CCS patients, while there was a positive correlation between IL‐6 and frailty in CCS patients (r_s_ = 0.291, *p *< 0.001).

## 4 DISCUSSION

### 4.1 The incidence of frailty in elderly patients is associated with CCS

Previous studies in Brazil, China, and Europe have shown that the overall incidence of frailty in senior citizens was 24%, 9.9%, and 7.7%, respectively.^20^, ^21^, ^22^ Our results showed that the prevalence of frailty among the studied elderly patients in China with CCS was 37.5%, thus suggesting that the prevalence of frailty was higher in elderly patients with CCS. Certain confirmation for our results comes from a cross‐sectional study in Eastern China^23^ where the prevalence of frailty in 208 elderly patients (≥60 years old) with CCS was 30.3%. This estimation was slightly lower than our results; we consider that the difference in the frailty assessment tools and the regional differences between Eastern and Western China could be a possible explanation for this difference. In addition, Ozmen et al.^4^ included 99 CCS patients aged ≥70 years in Turkey and found that the risk of adverse cardiac events in frailty patients was 3.48 times higher than that in non‐frailty patients, and the risk of death was 6.05 times higher than that in non‐frailty patients, suggesting that the prognosis of frailty in the elderly patients with CCS was worse. These data confirm our hypothesis that CCS and frailty are interrelated. We further suggest that the early screening and intervention of elderly people can greatly reduce the prevalence of frailty and then reduce the risk of adverse events in old patients with CCS.

### 4.2 Blood serum levels of IL‐6, Alb, and 25(OH)D could be prognostic factors for frailty in patients with CCS

A meta‐analysis of 23,910 old adults (including 32 cross‐sectional studies) showed that patients with frailty and pre‐frailty had higher levels of inflammation, indicated in the high blood serum levels of CRP and IL‐6, than those without frailty.^24^ Elevated serum levels of IL‐6 and CRP were closely related to muscle loss and reduced grip strength.^25^ High IL‐6 and CRP blood serum levels were also associated with a 40% higher risk of grip strength reduction that gradually increased two to three times 3 years after diagnosis.^26^ This study further reconfirmed the relationship between the blood serum levels of inflammatory factors and frailty. And though the blood serum levels of CRP was not an independent risk factor for frailty in our study, it was still statistically significant when the univariate analysis was conducted (*p *< 0.001). The reason for this difference may be that IL‐6 is located upstream of CRP in the inflammatory cascade, thus playing a broader role.^27^

According to a 3.5‐year follow‐up observation of elderly women in the community reported by Ferrucci et al., the increased IL‐6 blood serum levels were considered an important predictor of function loss, muscle strength, and motor ability decline in elderly women.^28^ It was found that IL‐6 directly stimulated muscle consumption by activating the ubiquitin–proteome pathway, thus destroying the cytoplasm and nucleoprotein in fibrocytes.^29^, ^30^ It indirectly lowered the levels of the growth hormone (GH) and insulin‐like growth factor‐1 (IGF‐1) and reduced protein synthesis, leading to sarcopenia.^31^ Ma et al. included 130 elderly patients and showed that the blood serum IL‐6 levels were negatively correlated with strength and the walking speed of frailty patients.^32^ After adjusting the data for parameters like age and gender, IL‐6 blood serum levels were also negatively correlated with the exercise tolerance of the elderly. Hence, the authors suggested that IL‐6 could be used as a biomarker of functional decline and frailty. In addition, other data linked the blood serum levels of IL‐6 with upcoming cardiovascular events in patients with coronary artery disease.^33^ Since IL‐6 plays an important role in both frailty and CAD, monoclonal antibodies blocking IL‐6 may be effective in clinical intervention of frailty in elderly patients with CCS. IL‐6 monoclonal antibody has not been reported in patients with frailty combined with CCS, although it is currently widely used in other areas.

In a study of 1368 subjects living in Tanushimaru, Japan, Yamamoto et al. found that lower albumin levels showed a linear trend with an indicator of frailty, namely the grip strength.^34^ The results of a cohort study showed that for every 1g/dL increase in the blood serum albumin concentrations, frailty scores decreased by 0.4 points, suggesting that patients with higher serum protein levels were at lower risk of frailty.^35^ Our study also showed that blood serum albumin concentrations in elderly patients with the frailty of CCS were significantly lower than that in pre‐frailty and non‐frailty patients. The multivariate logistic regression showed that the blood serum albumin was a protective factor for elderly patients with the frailty of CCS. Therefore, we speculate that serum albumin may be a biomarker of frailty in elderly patients with CCS. According to another prospective cohort study, changes in the blood serum protein concentrations are associated with various inflammatory indicators, especially when CRP and IL‐6 were analyzed.^36^ To date, the specific mechanism underlying this finding is not completely clear. Previous studies have suggested that IL‐6 induced increased vascular permeability and led to the diffusion of albumin into the extravascular fluid, resulting in a decrease in the blood serum protein concentrations.^34^

Serum albumin has long been considered an indicator of nutritional status.^37^, ^38^ A meta‐analysis of 5447 community‐based older adults at the mean age of 77.2 years showed a significant association between frailty and the prevalence of malnutrition (*p *< 0.001), and about 90% of all malnourished patients suffered from frailty.^39^ Lower protein intake, especially when accompanied by less physical activity, led to decreased muscle function, weakened muscle strength, slower walking speed, and fatigue.^40^, ^41^ It is generally accepted that protein supplementation can reduce the concentration of circulating muscle‐derived IL‐6 and can weaken the response of IL‐6 to muscle tissue injury, thus reducing the risk of frailty.^42^ Although protein supplementation is used to reduce the risk of frailty, there is no clear definition of the type, mode, and dose of this supplementation. Moreover, most studies are carried out in relatively healthy white individuals, while there are not enough reports on elderly Asians with CCS and frailty.

25(OH)D deficiency is a factor of poor prognosis in patients with CHD.^43^ With the decrease of 25(OH)D levels, the incidence of coronary heart disease and its severity increase, suggesting that 25(OH)D level can be used as an indicator to evaluate the severity of coronary heart disease.^43^, ^44^ Meanwhile, 25(OH)D is also closely related to frailty. Low serum levels of 25(OH)D were independently associated with frailty and frailty phenotype, according to the results of an Australian study.^45^ In addition, Tajar et al. confirmed that low 25(OH)D level was not only independently associated with frailty, but also associated with reduced walking speed, low physical activity, and fatigue in the frailty phenotype.^46^ Although there is a close relationship between 25(OH)D and frailty, their specific mechanism is not completely clear. Most studies believe that it is mediated by sarcopenia. Vitamin D receptor (VDR) acts on the principle of a nuclear receptor–mediated gene effect but also through non‐nuclear receptor‐mediated non‐gene effect could affect the proliferation and differentiation of muscle cells and protein synthesis.^47^ When vitamin D is deficient, its binding force with its receptor is weakened, resulting in decreased muscle mass and strength.^48^

25(OH)D plays an important role in a variety of physiological processes, such as musculoskeletal development and maintenance, emotional regulation, and autoimmunity.^49^, ^50^ 25(OH)D deficiency has been proved to be associated with an increased prevalence of frailty and the risk of CVD, as well as with elevated serum IL‐6 levels.^51^, ^52^, ^53^ Therefore, we speculate that 25(OH)D may be a predictor of frailty in elderly patients with CCS, while vitamin D supplementation may have a good therapeutic effect on them. However, supplementation with this vitamin proved to lead to little effect on inflammation, muscle mass, or atherosclerotic heart disease, according to some studies.^54^, ^55^ The reason for this result may be due to the special constitution of the elderly patients, compared with ordinary adults, or because vitamin D supplementation may require longer time or larger doses to have a beneficial effect. Moreover, the ideal concentrations of vitamin D and the period for supplementation need to be further studied and discussed.

## 5 CONCLUSIONS

The results of this study showed that the prevalence of frailty was 37.5% among elderly patients with CCS. We also confirm that CCS and frailty interact and promote each other and prompt that early screening and intervention can greatly reduce the prevalence of frailty and the risk of adverse events in elderly patients with CCS. Meanwhile, we found that the blood serum levels of IL‐6, Alb, and 25(OH)D were independently corrected with frailty in elderly patients with CCS, demonstrating that IL‐6 played as a risk factor for frailty in elderly patients with CCS, while Alb and 25(OH)D were protective factors. Therefore, serum IL‐6, Alb, and 25 (OH) D are expected to be predictive factors or objective biological indicators for the diagnosis of frailty in elderly patients with CCS. In addition, the supplementation with protein and vitamin D as well as the blockage of IL‐6 are expected to be a potential clinical intervention of frailty in elderly patients with CCS.

Three definite innovative features of our study can be highlighted. (1) the mobile software platform used to collect data for the evaluation of the frailty and related symptoms of elderly CCS patients completely replaced the traditional paper version and greatly reduced data collection time and statistical errors. It also increased the reliability of the data. (2) We screened for the first time the influencing factors of frailty patients with CCS extracted from general demographic data, geriatric syndrome, and serum biomarkers, and focused on the correlation between the blood serum concentrations of IL‐6, Alb, 25(OH)D, and frailty of elderly patients with CCS. (3) We proposed for the first time that blood serum levels of IL‐6, Alb, and 25(OH)D may be prognostic factors or objective biological indicators for the diagnosis of frailty in elderly patients with CCS. We further prosed that protein, vitamin D supplementation, and IL‐6 blockade could be potential methods for the clinical intervention of frailty in elderly patients with CCS.

There are also some limitations to our study. First, we excluded patients who could not cooperate with the comprehensive geriatric assessment, like critically ill patients, resulting in lower than actual morbidity levels. At the same time, in the diagnostic criteria of CCS, patients with stable symptoms within 1 year after ACS or who have received revascularization recently (within 1 year) are the main ones. Because this criterion is easy to identify and has a clear diagnosis, while other diagnostic criteria are relatively difficult. Therefore, relatively few subjects were included. Second, the sample size of this study was relatively small as it was a single‐center study with certain regional differences, which may lack high representativeness. Finally, it was a cross‐sectional study and could not directly investigate the causal relationship between serum IL‐6, Alb, 25(OH)D, and frailty in elderly patients with CCS. All these limitations exert the need for future investigations in the field.

## CONFLICT OF INTEREST

There is no conflict of interest in this article.

## AUTHOR CONTRIBUTIONS

Jing‐rong Dai was responsible for the conception and design of the paper, the analysis and interpretation of the results, as well as the writing of the paper. Yan Li carried out the implementation and feasibility analysis of the research and was responsible for the quality control and review of the paper. Data collection was done by Jie Li, Xu He, and Hong Huang. Xu He and Hong Huang sorted out and handled input data. Jie Li conducted the statistical processing and revised the paper. Yan Li was responsible for the supervision and management of the article.
